# Supplementary material for: Modulation of tonotopic ventral medial geniculate body is behaviorally relevant for speech recognition
Source: eLife. 2019 Aug 27;8:e44837. doi: 10.7554/eLife.44837 (PMC6711666; doi:10.7554/eLife.44837)
Supplement: Supplementary file 1. — Family-wise error corrected p-values were calculated using small volume correction for the voxels within the masks. [file elife-44837-supp1.docx]

Supplementary file 1. Montreal neurological institute (MNI) coordinates, p-values, T-values and parameter estimates (β) and 90% confidence intervals (CI) for voxels within regions for which we did not have an a-priori hypothesis. Family-wise error corrected p-values were calculated using small volume correction for the voxels within the masks.

| Mask | MNI coordinate | p-value (FWE) | T-value | β and 90% CI |
| --- | --- | --- | --- | --- |
| Speech vs. speaker | | | | |
| Left MGB gradient 2 | (-12, -27, -5) | 0.694 | -1.95 | -1.50 [-2.77, -0.24] |
| Right MGB gradient 1 | (12, -28, -2) | 0.756 | 0.25 | 0.17 [-0.95, 1.29] |
| Right MGB gradient 2 | (13, -27, -3) | 0.805 | 0.31 | 0.19 [-0.84, 1.23] |
| Left IC | (-4, -35, -9) | 0.109 | -2.71 | -1.58 [0.62, 2.54] |
| Right IC | (7, -35, -11) | 0.357 | -1.97 | -1.36 [-2.49, -0.22] |
| Speech vs. speaker correlated with proportion of hits in speech task. | | | | |
| Left MGB gradient 2 | (-12, -28, -5) | 0.223 | 2.24 | 1.03 [0.22, 1.83] |
| Right MGB gradient 1 | (13, -28, -3) | 0.126 | 2.52 | 0.77 [0.27, 1.27] |
| Left IC | (-2, -36, -12) | 0.211 | 2.43 | 0.58 [0.19, 0.98] |
| Right IC | (4, -36, -11) | 0.315 | 2.08 | 0.59 [0.12, 1.06] |
