# Supplementary material for: Modulation of tonotopic ventral medial geniculate body is behaviorally relevant for speech recognition
Source: eLife. 2019 Aug 27;8:e44837. doi: 10.7554/eLife.44837 (PMC6711666; doi:10.7554/eLife.44837)
Supplement: Transparent reporting form [file elife-44837-transrepform.pdf]

eLife's transparent reporting form

We encourage authors to provide detailed information *within their submission* to facilitate the interpretation and replication of experiments. Authors can upload supporting documentation to indicate the use of appropriate reporting guidelines for health-related research (see EQUATOR Network), life science research (see the BioSharing Information Resource), or the ARRIVE guidelines for reporting work involving animal research. Where applicable, authors should refer to any relevant reporting standards documents in this form.

If you have any questions, please consult our Journal Policies and/or contact us: editorial@elifesciences.org.

Sample-size estimation

- You should state whether an appropriate sample size was computed when the study was being designed
- You should state the statistical method of sample size computation and any required assumptions
- If no explicit power analysis was used, you should describe how you decided what sample (replicate) size (number) to use

Please outline where this information can be found within the submission (e.g., sections or figure legends), or explain why this information doesn't apply to your submission:

There are three previous studies that show the effect of enhanced BOLD response for a speech recognition task in contrast to control tasks on the same stimulus input. These previous studies used sample sizes between 14 and 17 participants (von Kriegstein et al. 2008; Díaz et al., 2012). For the present study we doubled the sample size to 33 participants to ensure sufficient power to detect the effect.

For the tonotopic localiser a previous study used a sample of 6 participants (Moerel et al., 2015). We estimated a sample of 28 participants to have enough power to create the tonotopic map.

VON KRIEGSTEIN, K., PATTERSON, R. D. & GRIFFITHS, T. D. 2008. Task-dependent modulation of medial geniculate body is behaviorally relevant for speech recognition. *Curr Biol,* 18**,** 1855-9.

DÍAZ, B., HINTZ, F., KIEBEL, S. J. & KRIEGSTEIN, K. V. 2012. Dysfunction of the auditory thalamus in developmental dyslexia. *Proceedings of the National Academy of Sciences,* 109**,** 13841-13846.

MOEREL, M., DE MARTINO, F., UĞURBIL, K., YACOUB, E. & FORMISANO, E. 2015. Processing of frequency and location in human subcortical auditory structures. *Scientific Reports,* 5.

Replicates

- You should report how often each experiment was performed
- You should include a definition of biological versus technical replication
- The data obtained should be provided and sufficient information should be provided to indicate the number of independent biological and/or technical replicates
- If you encountered any outliers, you should describe how these were handled
- Criteria for exclusion/inclusion of data should be clearly stated

- High-throughput sequence data should be uploaded before submission, with a private link for reviewers provided (these are available from both GEO and ArrayExpress)

Please outline where this information can be found within the submission (e.g., sections or figure legends), or explain why this information doesn't apply to your submission:

We performed the experiment once (mentioned in Materials and Methods subsection Experiments). We did not encounter outliers and did not exclude any data (mentioned in Materials and Methods, Participants).

Statistical reporting

- Statistical analysis methods should be described and justified
- Raw data should be presented in figures whenever informative to do so (typically when N per group is less than 10)
- For each experiment, you should identify the statistical tests used, exact values of N, definitions of center, methods of multiple test correction, and dispersion and precision measures (e.g., mean, median, SD, SEM, confidence intervals; and, for the major substantive results, a measure of effect size (e.g., Pearson's r, Cohen's d)
- Report exact p-values wherever possible alongside the summary statistics and 95% confidence intervals. These should be reported for all key questions and not only when the p-value is less than 0.05.

Please outline where this information can be found within the submission (e.g., sections or figure legends), or explain why this information doesn't apply to your submission:

Statistical analyses are reported in the Materials and Methods (subsection Data Acquisition and Processing for behavioral and fMRI data). Statistical reporting of results can be found in the Results section for both behavioral and fMRI data.

(For large datasets, or papers with a very large number of statistical tests, you may upload a single table file with tests, Ns, etc., with reference to sections in the manuscript.)

Group allocation

- Indicate how samples were allocated into experimental groups (in the case of clinical studies, please specify allocation to treatment method); if randomization was used, please also state if restricted randomization was applied
- Indicate if masking was used during group allocation, data collection and/or data analysis

Please outline where this information can be found within the submission (e.g., sections or figure legends), or explain why this information doesn't apply to your submission:

This information does not apply to our submission, as we only had one group.

Additional data files ("source data")

- We encourage you to upload relevant additional data files, such as numerical data that are represented as a graph in a figure, or as a summary table

eLife Sciences Publications, Ltd is a limited liability non-profit non-stock corporation incorporated in the State of Delaware, USA, with company number 5030732, and is registered in the UK with company number FC030576 and branch number BR015634 at the address 1st Floor, 24 Hills Road, Cambridge CB2 1JP | August 2014

- Where provided, these should be in the most useful format, and they can be uploaded as "Source data" files linked to a main figure or table
- Include model definition files including the full list of parameters used
- Include code used for data analysis (e.g., R, MatLab)
- Avoid stating that data files are "available upon request"

Please indicate the figures or tables for which source data files have been provided:

Behavioral data used to analyze and plot Figure S1 in Supplementary File 1 is provided in the Source Data File. Furthermore, we uploaded unthresholded maps to neurovault.org for the contrasts Speech vs. Speaker and the correlation Speech vs Speaker with proportion of hits in the speech task (Figure 5). Additionally, the tonotopic maps for left and right MGB are also provided (Figures 2 & 3), as well as the left vMGB mask (Figure 5). Files c3an be found at: https://neurovault.org/collections/CSGJQQDW/
